# Supplementary material for: Patterns of Intron Gain and Loss in Fungi
Source: PLoS Biol. 2004 Nov 30;2(12):e422. doi: 10.1371/journal.pbio.0020422 (PMC532390; doi:10.1371/journal.pbio.0020422)
Supplement: Table S1 — Also available at http://genes.mit.edu/NielsenEtAl/. (4.3 MB ZIP). [file pbio.0020422.st001.zip › NielsenEtAl/html/931.html]

AN0329.1.NCU06856.1.MG03175.1.FG06255.1


```
 CLUSTAL W (1.82) Multiple Sequence Alignments - Introns Inserted


Sequence 1: NCU06856.1	382 aa
Sequence 2: MG03175.1	379 aa
Sequence 3: FG06255.1	380 aa
Sequence 4: AN0329.1	393 aa
Alignment Length: 451 aa
Number Identitical Residues: 151 aa
Alignment Score (without introns) 8027


MG03175.1 	M~YA------------SAMR---GGRRPIVQRFDEYYRCYPMIMVPGAERPELNHGSKII
NCU06856.1	M0YG------------DIYSRA-TGRSRGAQRFDEYYRCYPLVMAPGAERPELNYGSKIL
FG06255.1 	M0YSGFGGGYDPANPEHLYNMARHGRRPIVQRFDEYYRCYPLVMAPGAERPELNYGSKII
AN0329.1  	-~------------------------------------------MPGPERENVNHGGKVI
          	                                             **.** ::*:*.*::

MG03175.1 	LPPSALEKVSKLHVQWPLLMELINGENDKHTHSGVLEFVAEEGRAYLPQW~MMQTLQLDV
NCU06856.1	LPPSALDKVSRLHVQWPIMLELINGSQGTHTHAGVLEFVAEEGRAYIPQW~MMQTLKLDV
FG06255.1 	LPPSALDKVSKLHVQWPLLMELINGEKGRHSHAGVLEFIAEEGRAYIPQW0MMVTLGMDV
AN0329.1  	MPPSALDKLTRLHITYPMLFELHNGAKEKMSHAGVLEFIAEEGKIYLPYW0LMQTLLLEP
          	:*****:*:::**: :*:::** ** :   :*:*****:****: *:* * :* ** :: 

MG03175.1 	GDMIQIKSTSLELARMVKLQPQSAKFLDISDPRAVLEKAFRNFATLTKGDVFSFEYNDDV
NCU06856.1	GDMIQIKTTSLELARLVKLQPQSVNFLDISDPRAVLEKAFRNFAALTKGDVFNFEYNDEI
FG06255.1 	GDMIQIRTTSLELAKMVKLQPQSVNFLEISDPKAVLEKAFRNFATLTKGDVFNFSYNDEV
AN0329.1  	GDLVQIKSTDLPPGRFIKLQAQSTSFLDISDPKAVLENAFRNFSCLTKDDVFTFAYNDQV
          	**::**::*.*  .:::***.**..**:****:****:*****: ***.***.* ***::

MG03175.1 	YEMAVLDVKPETTKMGVSMIETDVSVDFAAPVGYVEPERQAKGSGTSTPRSTR--GGGLP
NCU06856.1	YEMAVLDVKPETAKMGVCMIETDVSVDFAPPVGYVEPSRQTQGSGTSTPRNGRAVGGGLP
FG06255.1 	YDVAVLDVKPETDKMGVSMIETDVSVEFAPPVGYVEPERQ---SGTSTPRSTR---GGAP
AN0329.1  	YEMAVLETKPANDTNAISVLETDLEVDFAPPVGYEEPQRP---SGTSTPGSVVS--GRLP
          	*::***:.** . . .:.::***:.*:**.**** **.*    ****** .  :  *  *

MG03175.1 	AGGLLHSQGGMAQAINYDAIAPGSARSAA------TNFMGEGNKIGSKKGSKDGTPVKSS
NCU06856.1	AGGLLHNQGTMAQAINYDAIAPGATTTSV------GNFQGEGLRLAAKKGSKTGTP-KPA
FG06255.1 	AGGLLHNQGTMAQSINYSAIAP-SVTSNV------TNFLGEGQKL-VKKSSKASTP-KPA
AN0329.1  	AGGLLHPHGTMAQSINYAAIAPESTDAAAGARAVSSNFLSGGHRLNAKKGSKTPTP-TAS
          	****** :* ***:*** **** :. : ..: : : ** . * ::  **.**  ** ..:

MG03175.1 	TPVAGVSTNPNTSGTSQDLIPRRRTN-GPLPLRLPANKLFFGYEIKPVKTDAQKEEEKES
NCU06856.1	TPVAGTSTN-----VKDVVLPRRKNNNGPLPLRLPPNKLFLGYEIKPVKTAADKEREAAA
FG06255.1 	TPVPTVDIPG---------MPKRRDG-APAALRLAPNKLFFGYEIKPVKTDADKEKEKES
AN0329.1  	TPTPGATNPS-------HPPPVKRTN-GPQPLRLPPNQLFFGYAIKPVPKRDEGGNVVE-
          	**.. .              * :: . .* .***..*:**:** **** .  :  .    

MG03175.1 	AKRPHFAGQGQTLRGGAAK~RKG--------------~------DENEQLPTPEKRPGE-
NCU06856.1	AKQPHFAGQGQTLRGGVPK~KKSS-------------~------DGDDKAAQGEKKPTES
FG06255.1 	ANRPHFAGQGQTLRG-AVK~RKG--------------~------EGDDKTKAPEKKGTS-
AN0329.1  	PEKPRFQGTGQSLRGKKKE1AIAGRSCRFEYSPDRDF1IDFQPTTSKQQLTPIAAPPITA
          	.::*:* * **:***   :   .. :.  . :.. .   . ..:  .::          :

MG03175.1 	-GRRLDGKT----------~----------------
NCU06856.1	KGRRLDGRNV---------~----------------
FG06255.1 	EGNRLDGRRPR--------~----------------
AN0329.1  	SGFQLTRWAMDTSMSLSQK0LIFARLPFVRYYKKRS
          	.* :*      :: : :..    :  .     .. :
```
